# Editorial: PVT1 in Cancer

**DOI:** 10.3389/fonc.2020.588786

**Published:** 2020-10-20

**Authors:** Olorunseun O. Ogunwobi, Miguel F. Segura

**Affiliations:** ^1^Department of Biological Sciences, Hunter College of The City University of New York, New York, NY, United States; ^2^Laboratory of Translational Research in Child and Adolescent Cancer, Hospital Universitari Vall d'Hebron, Vall d'Hebron Institut de Recerca, VHIR, Universitat Autònoma de Barcelona, Barcelona, Spain

**Keywords:** PVT1, cancer, long non-coding RNA, non-coding RNA, chromosome 8q24

The plasmacytoma variant translocation 1 (PVT1) gene is located at human chromosome 8q24, downstream of the well-known c-MYC oncogene (1). As chromosome 8q24 is a chromosomal region of genomic instability, it is not surprising that PVT1 was discovered in the context of cancer. PVT1 is now known to be dysregulated in non-cancerous diseases such as kidney disease (including diabetic nephropathy) (2), cardiac hypertrophy (3), vitiligo (4), osteoarthritis (5), and asthma (6). However, PVT1 is much better established to be dysregulated in a wide variety of cancers including plasmacytomas (7, 8), lymphomas (9, 10), leukemias (11, 12), sarcomas (including osteosarcoma) (13, 14), ovarian cancer (15, 16), breast cancer (16, 17), lung cancer (18, 19), astrocytomas (20), pancreatic cancer (21, 22), prostate cancer (23–26), cholangiocarcinoma (27), gliomas (28), medulloblastoma (29), mesothelioma (30), colorectal cancer (31), gastric cancer (32), hepatocellular carcinoma (33, 34), thyroid cancer (35), bladder cancer (36), renal cell carcinoma (37, 38), cervical cancer (39), esophageal cancer (40), melanoma (41), endometrial cancer (42, 43), non-small cell lung cancer (44, 45), and cutaneous squamous cell carcinoma (46, 47).

PVT1 has at least 12 annotated exons: exon 1A, exon 1B, exon 1C, exon 2, exon 3A, exon 3B, exon 4A, exon 4B, exon 5, exon 6, exon 7, exon 8, and exon 9 (1). And it encodes six annotated microRNAs (miRNAs): miR-1204, miR-1205, miR-1206, miR-1207-3p, miR-1207-5p, and miR-1208 (48). PVT1 is expressed in the various organs throughout the human body. There is progressively increasing evidence that distinct PVT1 exons, and PVT1-encoded miRNAs have significant biological functions, as discussed in the well-written articles included in our Research Topic entitled “PVT1 in Cancer.” In addition, there is evidence of alternative splicing at the PVT1 gene, resulting in at least 25 annotated PVT1 transcript variants (Martinez-Barriocanal et al.). As noted in several of the papers published in the Research Topic “PVT1 in Cancer,” PVT1 induces cancer development and progression via a variety of biological mechanisms including but not limited to miRNA regulation (Wang et al.), and as a competing endogenous RNA (ceRNA) (Ogunwobi and Kumar).

The articles included in the Research Topic “PVT1 in Cancer” are particularly interesting because they highlight the clinical relevance and potential clinical applications of PVT1 in cancer. For example, the article by Boloix et al. discusses the potential prognostic applications and the potential to target PVT1 for therapeutic applications in pediatric cancers. All of the other articles discuss potential clinical applications in a variety of adult cancers. Notably, Ogunwobi and Kumar identify PVT1 as a mediator of cancer chemoresistance. Thus, targeting PVT1 may have a future role in the treatment of many highly lethal cancers such as pancreatic cancer, and neuroendocrine prostate cancer where chemoresistance is common.

## Author Contributions

All authors listed have made a substantial, direct and intellectual contribution to the work, and approved it for publication.

## Conflict of Interest

OO is a co-founder of NucleoBio, Inc., a City University of New York start-up biotechnology company. The remaining author declares that the research was conducted in the absence of any commercial or financial relationships that could be construed as a potential conflict of interest.
